# The 100 most cited articles investigating the radiological staging of oesophageal and junctional cancer: a bibliometric analysis

**DOI:** 10.1007/s13244-016-0505-6

**Published:** 2016-06-08

**Authors:** Kieran G. Foley, Arfon Powell, Wyn G. Lewis, Stuart Ashley Roberts

**Affiliations:** Division of Cancer & Genetics, Cardiff University, Cardiff, UK; Department of Surgery, University Hospital of Wales, Cardiff, UK; Department of Radiology, University Hospital of Wales, Cardiff, UK

**Keywords:** Bibliometric analysis, Oesophageal cancer, Gastro-oesophageal junction cancer, TNM staging, Citation

## Abstract

**Objectives:**

Accurate staging of oesophageal cancer (OC) is vital. Bibliometric analysis highlights key topics and publications that have shaped understanding of a subject. The 100 most cited articles investigating radiological staging of OC are identified.

**Methods:**

The Thomas Reuters Web of Science database with search terms including “CT, PET, EUS, oesophageal and gastro-oesophageal junction cancer” was used to identify all English language, full-script articles. The 100 most cited articles were further analysed by topic, journal, author, year and institution.

**Results:**

A total of 5,500 eligible papers were returned. The most cited paper was Flamen et al. (n = 306), investigating the utility of positron emission tomography (PET) for the staging of patients with potentially operable OC. The most common research topic was accuracy of staging investigations (n = 63). The article with the highest citation rate (38.00), defined as the number of citations divided by the number of complete years published, was Tixier et al. investigating PET texture analysis to predict treatment response to neo-adjuvant chemo-radiotherapy, cited 114 times since publication in 2011.

**Conclusion:**

This bibliometric analysis has identified key publications regarded as important in radiological OC staging. Articles with the highest citation rates all investigated PET imaging, suggesting this modality could be the focus of future research.

***Main Messages*:**

• *This study identifies key articles that investigate radiological staging of oesophageal cancer*.

• *The most common topic was accuracy of staging investigations.*

• *The article with the highest citation rate investigated the use of texture analysis in PET images*.

## Introduction

Bibliometric analysis assesses the number of times that an article is cited in the literature, and in which particular journal. A citation is received when an article references another peer-reviewed publication. An article that is felt to have greater importance and higher impact by the scientific community is more likely to be cited and therefore may be more influential on current healthcare practice. Articles and journals can be ranked based on the number of citations they receive. Bibliometric analysis also reveals topics of current interest, identifies potential novel techniques and shows historical developments in that subject [1]. Medical researchers have used bibliometric analysis to identify the most influential papers in their clinical specialties, including orthopaedic surgery [2] and oncology [3].

Worldwide, the prognosis of oesophageal cancer, including gastro-oesophageal junction cancer (OC), is poor. Overall 5-year survival in the UK is approximately 13 % [4]. As a part of the diagnostic pathway, patients undergo a variety of staging investigations to assess the extent of disease. Radiological staging is performed to further inform management decisions by the multi-disciplinary team (MDT). Accurate radiological staging is vital to ensure the most appropriate treatment is selected. Currently, OC is staged according to the International Union Against Cancer (UICC) Tumour Node Metastasis (TNM) 7th edition [5].

In the UK, patients with OC are initially staged with CT of the thorax and abdomen to exclude irresectable disease or distant metastases. If the patient is deemed suitable for radical treatment, either in the form of definitive chemo-radiotherapy (dCRT) or surgery (± neo-adjuvant therapy), positron emission tomography combined with computed tomography (PET/CT) and endoscopic ultrasound (EUS) are performed for a more detailed assessment of disease stage [6].

This bibliometric analysis of OC staging investigations aims to identify key research that has influenced staging methods, the institutions leading this research, studies that may change staging methods in the future and imaging modalities being focused upon.

## Materials and methods

The Thomas Reuters Web of Science citation indexing database was used to perform the search. The following search terms were used in order to capture the variety of imaging modalities and the different nomenclature of tumours: (oesophag* AND (neoplas* OR cancer* OR carcin* OR tumo* OR malig*)) OR (esophag* AND (neoplas* OR cancer* OR carcin* OR tumo* OR malig*)) OR (gastro-oesophageal junction AND (neoplas* OR cancer* OR carcin* OR tumo* OR malig*)) OR (oesophago-gastric junction AND (neoplas* OR cancer* OR carcin* OR tumo* OR malig*) AND (computed tomography OR CT OR CAT) OR (positron emission tomography OR PET OR F18 OR FDG OR fluorodeoxyglucose) OR (endoscopic ultrasonography OR endoscopic ultrasound OR endosonographic OR EUS) OR (magnetic resonance imaging OR MRI or diffusion weight* OR DWI) AND (stag* OR TNM OR lymph node OR metasta*). The search was performed on 18 September 2015.

All databases within the Thomas Reuters Web of Science were searched. The results were filtered to include only full script articles written in the English language, throughout all available years. The results were sorted by number of citations, with the article with most citations analysed first. The method was developed by Paladugu et al. [7].

The title and abstract of the returned articles were manually assessed to ensure that their relevance and content were in keeping with this field. The inclusion criterion was that the article investigated the use of a single or combination of radiological modalities in patients with OC. This criterion was pre-specified and defined prior to data collection.

Articles were excluded from the list if the content was not relevant to radiological OC staging. The 100 most cited articles were identified and further analysed.

The articles were further evaluated for the publishing journal, names of the first and senior author, the institution and department to which the first author was affiliated, the country of origin, year of publication, the radiological investigation(s) being studied, the topic of the article and the number of citations according to Web of Science. Rank within the top 100 articles was also recorded.

Articles have the opportunity to accrue more citations if they have been published for longer. To adjust for this, a citation rate was calculated, defined as the number of citations divided by the number of complete years published. A list of the ten articles with the highest citation rates is provided.

In addition, the individual and 5-year impact factors in 2014 were recorded for the publishing journal. The overall median 2014 and 5-year impact factor for all journals was calculated.

## Results

The Web of Science search returned 5,500 full articles, written in English language. The 100 most cited articles are listed in Table 1 [8–107].

**Table 1:** The 100 most cited articles in radiological staging of oesophageal and junctional cancer

Rank	Number of Citations	First author
1	306	Flamen P [8]
2	294	Tio TL [9]
3	290	Kinkel K [10]
4	273	Catalano MF [11]
5	271	Wieder HA [12]
6	261	Botet JF [13]
7	242	Skinner DB [14]
8	229	Ott K [15]
9	221	Bhutani MS [16]
10	201	Flamen P [17]
11	193	Downey RJ [18]
12	192	Flanagan FL [19]
13	189	Picus D [20]
14	184	Rosch T [21]
15	183	Kelly S [22]
16	168	van Westreenen HL [23]
17	165	May A [24]
18	164	Lerut T [25]
19	160	Kato H [26]
20	159	Block MI [27]
20	159	Swisher SG [28]
22	157	Swisher SG [29]
23	150	Luketich JD [30]
24	146	Rice TW [31]
24	146	Vilgrain V [32]
26	145	van Vliet EPM [33]
27	143	Rosch T [34]
28	137	Ziegler K [35]
29	136	Kole AC [36]
30	135	Luketich JD [37]
31	133	Buskens CJ [38]
32	132	Watt I [39]
33	130	Moss AA [40]
34	123	Zuccaro G [41]
35	122	Grimm H [42]
36	121	Vazquez-Sequeiros E [43]
37	116	Yoon YC [44]
38	115	Tio TL [45]
39	114	Cerfolio RJ [46]
39	114	Tixier F [47]
41	113	Vazquez-Sequeiros E [48]
42	112	Dittler HJ [49]
43	111	Rasanen JV [50]
44	108	Quint LE [51]
45	107	Reed CE [52]
46	105	Choi JY [53]
46	105	Rankin SC [54]
46	105	Thompson WM [55]
46	105	Wallace MB [56]
50	104	Quint LE [57]
51	103	Kato H [58]
51	103	Larghi A [59]
53	102	Takashima S [60]
54	100	van Westreenen HL [61]
55	98	Eloubeidi MA [62]
55	98	Hasegawa N [63]
57	96	Levine EA [64]
57	96	Rice TW [65]
59	92	Isenberg G [66]
59	92	Leong T [67]
59	92	Puli SR [68]
62	91	Flamen P [69]
62	91	Lightdale CJ [70]
64	89	Daffner RH [71]
64	89	Kim K [72]
64	89	Meltzer CC [73]
64	89	Vrieze O [74]
68	88	Jones DR [75]
69	86	Chak A [76]
70	84	Hyun SH [77]
70	84	Quint LE [78]
72	83	Murata Y [79]
73	82	Hiele M [80]
74	80	Wakelin SJ [81]
75	79	Scotiniotis IA [82]
76	78	Fockens P [83]
77	76	Beseth BD [84]
77	76	Catalano MF [85]
77	76	Giovannini M [86]
80	75	Rice TW [87]
81	74	Heeren PAM [88]
81	74	Rizk N [89]
83	72	Kostakoglu L [90]
83	72	Moureau-Zabotto L [91]
85	70	Pech O [92]
86	69	Choi JY [93]
86	69	Lehr L [94]
86	69	Lightdale CJ [95]
89	68	Kobori D [96]
89	68	Luketich JD [97]
91	67	Lowe VJ [98]
92	62	Song SY [99]
92	62	Yuan S [100]
94	61	Bar-Shalom R [101]
94	61	McAteer D [102]
94	61	van Westreenen HL [103]
97	60	Eloubeidi MA [104]
97	60	Konski A [105]
99	59	Meyers BF [106]
100	46	Wu LF [107]

The article with the highest number of citations (n = 306) was Flamen et al. [8], entitled ‘Utility of positron emission tomography for the staging of patients with potentially operable oesophageal carcinoma’. The article ranked 100 in the list was Wu et al. [107], entitled ‘Preoperative TN staging of oesophageal cancer: comparison of miniprobe ultrasound, spiral CT and MRI’, with 46 citations.

The oldest article was published in 1979 by Daffner et al. [71] ‘CT of the Oesophagus. 2. Carcinoma’. Tixier et al. [47] published the most recent paper in the list in 2011, entitled ‘Intratumor Heterogeneity Characterized by Textural Features on Baseline F-18-FDG PET Images Predicts Response to Concomitant Radiochemotherapy in Esophageal Cancer’, which has been cited 114 times.

The journal with the highest number of published articles was Gastrointestinal Endoscopy (Table 2). Fourteen articles were published with a total of 1675 citations [11, 16, 34, 38, 48, 59, 62, 63, 66, 76, 80, 82, 83, 85]. The 2014 impact factor of the Gastrointestinal Endoscopy was 5.369, with 5-year impact factor 5.225. The journal with the highest impact factor was the Journal of Clinical Oncology (JCO), which had a total of 1,258 citations [8, 12, 15, 18, 23, 70]. Five of these six articles were investigating PET imaging. The 2014 impact factor of JCO was 18.428, with 5-year impact factor 16.996. Overall, the median 2014 impact factor of the journals was 5.238 and median 5-year impact factor was 5.225.

**Table 2:** Journals with ≥2 articles in 100 most cited

Journal	Number of articles	2014 Impact factor	5-Year impact factor	Total number of citations
Gastrointestinal Endoscopy	14	5.369	5.225	1,675
Annals of Thoracic Surgery	9	3.849	4.104	1,038
Journal of Nuclear Medicine	8	6.16	6.280	657
Cancer	7	5.238	5.517	830
American Journal of Roentgenology	6	2.731	3.302	764
Journal of Clinical Oncology	6	18.428	16.966	1,258
Radiology	6	6.867	7.259	1,044
Endoscopy	5	5.053	4.855	494
Journal of Thoracic and Cardiovascular Surgery	5	4.168	4.068	428
Annals of Surgery	3	8.327	8.844	502
Gut	3	14.66	12.553	485
International Journal of Radiation Oncology Biology Physics	3	4.258	4.359	194
American Journal of Gastroenterology	2	10.755	9.145	193
Annals of Surgical Oncology	2	3.93	4.532	195
British Journal of Cancer	2	4.836	5.305	281
Gastroenterology	2	16.716	13.811	415
Radiotherapy and Oncology	2	4.363	4.502	181
World Journal of Gastroenterology	2	2.369	2.671	138

Twenty-nine of the 100 articles were published in a radiology-related journal, including nuclear medicine and radiation oncology journals. Thirty-five of the first authors were affiliated to a radiology, nuclear medicine or radiation oncology department, according to the Thomas Reuters Web of Science citation indexing database. Three radiology-related journals, with 5-year impact factor >5.00, published 16 articles in the top 100. These were Radiology (5-year impact factor 7.259; n = 6), Journal of Nuclear Medicine (5-year impact factor 6.280; n = 8) and European Journal of Nuclear Medicine and Molecular Imaging (5-year impact factor 5.090; n = 1).

Researchers from the USA published the greatest number of articles in the 100 most cited (n = 47) [11, 13, 14, 16, 18–20, 27–31, 37, 40, 41, 43, 46, 48, 51, 52, 55–57, 59, 62, 64–66, 68, 70, 71, 73, 75, 76, 78, 82, 84, 85, 87, 89, 90, 95, 97, 98, 104–106], jointly followed by Germany [12, 15, 21, 24, 34, 35, 42, 49, 92, 94] and The Netherlands [9, 23, 33, 36, 38, 45, 61, 83, 88, 103] (n = 10, each) (Table 3). The Technical University of Munich, Germany, was the institution with the joint highest number of publications in the 100 Most Cited (n = 6) and the highest number of citations (1,008) [12, 15, 21, 34, 49, 94]. The University Hospital Gasthuisberg, Leuven, Belgium, also had 6 published articles, with a total of 933 citations [8, 17, 25, 69, 74, 80]. The most cited article was from this institution [8] and written by Dr Patrick Flamen (first author) with Prof. Luc Mortelmans as senior author. Dr Flamen has 3 first author articles in the 100 most cited [8, 17, 69] and a total of 598 citations. Prof. Mortelmans has 4 articles published as senior author [8, 17, 25, 69] and a total of 762 citations.

**Table 3:** Number of articles per country of origin in 100 most cited

Country	Total number of articles
USA	47
Germany	10
The Netherlands	10
Belgium	6
Japan	6
South Korea	6
UK	5
France	4
China	2
Australia	1
Finland	1
Israel	1
Switzerland	1

The most common researched topic was the accuracy of radiological staging investigations (n = 63) (Table 4). Several of the study themes overlapped but accuracy of staging was commonly compared between different modalities (n = 29). The investigation of lymph node metastases (n = 15) and radiological response to treatment (n = 14) were also commonly cited topics.

**Table 4:** Most frequently cited topics of investigation (numbers do not add up to 100 as there are different combinations of topics in the articles)

Topic	Number of articles
Accuracy of staging	63
Comparison of imaging modalities	29
Lymph node metastases	15
Treatment response	14
Review of staging	9
Imaging features of malignancy	9
Prognosis	7
Distant metastases	5
Treatment planning	4
Early cancer	3
Cost-effectiveness	1
Restaging	1
Staging recurrent cancer	1
Correlation with tumour markers	1
Synchronous tumours	1
Texture analysis	1

EUS was the most common modality investigated (n = 51), with PET/CT (n = 48) and CT (n = 46) following. The combination of CT, EUS and PET/CT was commonly investigated together, which is the recommended staging pathway for potentially curable disease in the UK (n = 11) [8, 25, 28, 31, 50, 53, 88, 98, 105]. MRI (n = 5), bone scintigraphy (n = 2), PET alone (n = 1), EUS-FNA (n = 1), US (n = 1) and laparoscopic US (n = 1) were also cited.

The article with the highest citation rate (38.00) was Tixier et al. [47], published in 2011 and investigated texture analysis in OC. The ten articles with the highest citation rates were published between 2002 and 2011 and all involved investigation of PET images (Table 5). Four of the articles investigated treatment response [12, 15, 18, 47]. An international collaboration collecting data that informed the International Union Against Cancer (UICC) Tumour Node and Metastasis (TNM) 7th edition [31] had the second highest citation rate (36.50). Five of the ten articles with the highest citation rates were published in the Journal of Clinical Oncology, which had the highest impact factor (5-year 16.971).

**Table 5:** Ten articles with the highest citation rates

Rank	Year	Number of citations	Citation rate	First author	Senior author	Title	Journal
1	2011	114	38.00	Tixier F [47]	Visvikis D	Intratumour heterogeneity characterized by textural features on baseline 18 F-FDG PET images predicts response to concomitant radiochemotherapy in oesophageal cancer	Journal of Nuclear Medicine
2	2010	146	36.50	Rice TW [31]	Blackstone EH	Cancer of the Oesophagus and Esophagogastric Junction Data-Driven Staging for the Seventh Edition of the American Joint Committee on Cancer/International Union Against Cancer Cancer Staging Manual	Cancer
3	2006	229	28.63	Ott K [15]	Siewert JR	Metabolic imaging predicts response, survival, and recurrence in adenocarcinomas of the esophagogastric junction	Journal of Clinical Oncology
4	2004	271	27.10	Wieder HA [12]	Weber WA	Time course of tumour metabolic activity during chemoradiotherapy of oesophageal squamous cell carcinoma and response to treatment	Journal of Clinical Oncology
5	2002	290	24.17	Kinkel K [10]	Thoeni RF	Detection of hepatic metastases from cancers of the gastrointestinal tract by using noninvasive imaging methods (US, CT, MR imaging, PET): A meta-analysis	Radiology
5	2008	145	24.17	van Vliet EPM [33]	Siersema PD	Staging investigations for oesophageal cancer: a meta-analysis	British Journal of Cancer
7	2000	306	21.86	Flamen P [8]	Mortelmans L	Utility of positron emission tomography for the staging of patients with potentially operable oesophageal carcinoma	Journal of Clinical Oncology
8	2010	84	21.00	Hyun SH [77]	Kim BT	Prognostic value of metabolic tumour volume measured by 18 F-fluorodeoxyglucose positron emission tomography in patients with oesophageal carcinoma	Annals of Surgical Oncology
9	2003	193	17.55	Downey RJ [18]	Rusch V	Whole body (18)FDG-PET and the response of oesophageal cancer to induction therapy: Results of a prospective trial	Journal of Clinical Oncology
10	2004	168	16.80	van Westreenen HL [23]	Plukker JTM	Systematic review of the staging performance of 18 F-fluorodeoxyglucose positron emission tomography in oesophageal cancer	Journal of Clinical Oncology

## Discussion

OC is the eighth most common malignancy worldwide, resulting in around 400,000 deaths per annum [108]. This study demonstrates that accuracy of staging was the most frequently studied topic (n = 63) (Table 4). Accurate staging investigations are vital to inform appropriate treatment decisions, providing the best chance of survival for the patient whilst minimising harm from over- or under-treatment. The most cited article was Flamen et al. [8], which investigated the use of PET in potentially operable OC.

The OC staging pathway is complex, utilising various modalities with different strengths and weaknesses. PET/CT is superior to CT for detection of distant metastases and influences the change of MDT management decisions in up to 38 % of patients [109], whereas EUS is superior to CT for T-staging [110]. Comparison of techniques allows a modality to be tested against the perceived “gold-standard” staging investigation. This may reflect the desire for a simplified staging pathway with fewer investigations or the desire to increase evidence and awareness of a particular modality, thus introducing potential publication bias.

Influential articles are more likely to be cited by the scientific and clinical community. These citations form the basis of a journal’s impact factor. The impact factor quantifies the average number of citations per manuscript published within that journal during a specific time period. Therefore, journals with a higher impact factor are recognised as being of higher quality and more likely to contain influential articles.

Radiological OC staging appeals to specialist radiologists and other members of the upper gastro-intestinal (GI) cancer MDT, and its clinical impact is great. The overall median 2014 and 5-year impact factors were 5.238 and 5.225, respectively, demonstrating that this field of research, often producing novel results, in a specific cancer population is not likely to be published in high-impact journals. The Journal of Clinical Oncology (JCO) had the highest 5-year impact factor (16.971) of articles in the 100 most cited.

In total, only 29 of the 100 most cited articles were published in radiology-related journals. This could represent the desire to achieve publication in a high-impact journal. The majority of radiology-related journals have impact factors <5.00. Only 16 % of the top 100 articles were published in radiology-related journals with a 5-year impact factor >5.00 (Radiology, Journal of Nuclear Medicine and European Journal of Nuclear Medicine and Molecular Imaging). It may also reflect a lack of research conducted by radiologists, which is supported by evidence from a National Cancer Research Institute (NCRI) survey in 2012, which commented upon the lack of academic radiologists [111].

Many of the first authors (n = 65) are not affiliated to radiology departments, according to Thomas Reuters Web of Science citation indexing database. It is possible the authors work closely with a radiologist as part of the specialist Upper GI cancer MDT or have a clinical radiologist as a named co-author.

EUS was the most commonly investigated modality overall. This may be a reflection of the current reliance and importance of EUS for T and N staging, considered the current “gold standard” [110].

Despite EUS being the most frequently investigated modality, the ten articles with the highest citation rates all investigated functional PET imaging. The CT component of the PET/CT examinations provided attenuation correction for PET data. Many PET/CT topics of research are relatively novel and have been described in other types of cancer. One of these topics, texture analysis, is the subject of the article with the highest citation rate [47]. Novel subjects are less likely to be published in high-impact journals, but may well be considered influential and provide the catalyst for future research.

Four of the PET/CT articles with the highest citation rates [12, 15, 18, 47] investigated its use in assessing treatment response. There is significant interest in the capability of metabolic imaging to assess for early treatment response, but these techniques have not been standardised for use outside of clinical research studies [112]. PET/CT scanning is expensive, and costly research could potentially only produce marginal long-term benefits for patients. The paradox of healthcare is that innovation increases expense, rather than producing more cost-effective and efficient processes, as is the case in industry [113]. These articles however are likely to be highly influential in forthcoming years, as the use of PET/CT increases in cancer imaging.

This bibliometric analysis has a number of limitations. Citation rates can be misleading because of various biases, e.g. institutional, language or self-citation bias. Older articles tend to accrue more citations compared to newer research. We attempted to adjust for this by calculating a citation rate, which may provide information regarding the importance and potential influence that the research has. This in itself has limitations as the likelihood of citation rises with increasing numbers of published articles in peer-reviewed journals. Only articles written in English were included, which may have excluded some frequently cited research in other languages. Also, this study concentrated on radiological staging rather than other techniques such as endoscopy and laparoscopy.

The expanding volume of published literature has increased significantly over the past few decades. Between 1978 to 1985 and 1994 to 2001, the annual number of Medline articles increased by 46 %, particularly in the area of clinical research [114]. The annual rate of publication growth in PubMed Medline was 5.6 % between 1997 and 2006, equating to a “doubling time” of 13 years [115]. This may explain the higher citation rate of PET/CT compared to that of EUS, as PET/CT is a more recent innovation. Overall, there are now more articles published per annum compared to previous years. This would therefore potentially increase the citation rate as a matter of course, not necessarily reflecting higher importance.

As expected, the older articles only described CT and perhaps a review of the last 10 years of literature only may be more reflective of contemporary staging practice. Another limitation is that only the first and senior authors of the articles were included in the current analysis. It is possible that these authors contributed to other articles in this list, but would not have been counted during analysis. These authors may therefore be under-represented in terms of published article numbers and have had a greater influence on current OC staging.

Of the 29 articles comparing imaging techniques or modalities, 17 studies correlated imaging findings and histopathological diagnosis, widely regarded as the “gold standard”. Limitations exist in radiological studies that compare new findings against a potentially inaccurate alternative imaging test. In this current study, articles that did not compare against pathological results included those investigating radiotherapy planning techniques and the diagnosis of distant metastases. In these studies, tissue was not necessarily sampled. There are several reasons why pathological confirmation is not possible. These include patients undergoing non-surgical management, which is true of the majority of cases of OC, and in situations where it would be unethical to obtain tissue purely for research purposes, such as in patients with unequivocal distant metastases.

There are further limitations of studies comparing imaging findings to histopathological specimens. Comparison of pre-treatment imaging characteristics in patients receiving neo-adjuvant therapy prior to surgery can be inaccurate, as the chemotherapy or radiotherapy may alter the morphology of the tumour. In this situation, a direct comparison is often not possible.

## Conclusion

This bibliometric analysis describes the 100 most cited articles in the field of radiological OC staging investigations. Common topics of investigation include the accuracy of staging, comparison of modalities, treatment response and assessment of lymph nodes for metastases. The majority of articles are published outside of radiology-related journals, which may reflect the desire for high-impact publications or perhaps a lack of radiologists conducting imaging research. This study provides an understanding of research that has influenced current OC staging and citation rates may suggest important topics for future research, particularly validation studies of innovative techniques in larger patient populations.
